# Activity-dependent compensation of cell size is vulnerable to targeted deletion of ion channels

**DOI:** 10.1038/s41598-020-72977-6

**Published:** 2020-09-29

**Authors:** Srinivas Gorur-Shandilya, Eve Marder, Timothy O’Leary

**Affiliations:** 1 Volen Center and Biology Department, Brandeis University, Waltham, MA 02454 USA; 2 Department of Engineering, University of Cambridge, Cambridge, UK

**Keywords:** Biophysics, Cell biology, Computational biology and bioinformatics, Developmental biology, Neuroscience, Physiology

## Abstract

In many species, excitable cells preserve their physiological properties despite significant variation in physical size across time and in a population. For example, neurons in crustacean central pattern generators generate similar firing patterns despite several-fold increases in size between juveniles and adults. This presents a biophysical problem because the electrical properties of cells are highly sensitive to membrane area and channel density. It is not known whether specific mechanisms exist to sense membrane area and adjust channel expression to keep a consistent channel density, or whether regulation mechanisms that sense activity alone are capable of compensating cell size. We show that destabilising effects of growth can be specifically compensated by feedback mechanism that senses average calcium influx and jointly regulate multiple conductances. However, we further show that this class of growth-compensating regulation schemes is necessarily sensitive to perturbations that alter the expression of subsets of ion channel types. Targeted perturbations of specific ion channels can trigger a pathological response of the regulation mechanism and a failure of homeostasis. Our findings suggest that physiological regulation mechanisms that confer robustness to growth may be specifically vulnerable to deletions or mutations that affect subsets of ion channels.

## Introduction

In many animals, the physical size of neurons grows together with the body size of the animal, yet neural circuits continuously maintain their function. For example, action potential frequency and amplitude in Retzius neurons in the leech is constant over fourfold increase in cell diameter^1^. The A4I1 neuron in locust shows similar wind-sensitivity in first instar nymphs and in adults, despite a greater than threefold increase in cell size^2^. RPeD1, a neuron in the respiratory central pattern generator in *Lymnaea*, maintains resting membrane potential, spike amplitude and membrane resistance despite twofold growth from juvenile to adult^3^. In crickets, patterns of motor neuron output responsible for song production appear up to four moults before the adult form^4^. In lobsters, the co-ordinated bursting activity of pyloric neurons is indistinguishable between juveniles and adults, despite a many-fold increase in their size^5^. Even in embryonic stages, when these cells are even smaller, the pyloric circuit central pattern generator (CPG) expresses spontaneous albeit less stereotyped rhythmic activity^6,7^.

These widespread observations raise the question of how electrical properties of neural circuits remain intact during growth. Biophysical properties of neurons are heavily dependent on membrane area and ion channel density. It is not known whether specific mechanisms exist to sense membrane area and adjust channel expression to compensate changes in cell size, or whether other kinds of known regulation mechanisms can achieve this goal. Intriguingly, abnormal variations in cell sizes and presumed loss of cell size related excitability compensation have been implicated in disease processes. In cardiac cells, pathologies such as inexcitability and a reduction in resting membrane potential co-occur with structural abnormalities like cellular hypertrophy^8^. Purkinje cells can alter their size, possibly as a compensatory mechanism to restore electrophysiological function after injury, suggesting that loss of cell-size related excitability compensation can lead to disease processes^9^.

Activity-dependent regulation was first hypothesised^10^ and then experimentally demonstrated to maintain neuronal firing and other physiological properties by tuning neuronal parameters like ion channel densities^11–16^. Such regulation mechanisms employ feedback control of channel expression using intracellular signals, most notably calcium concentration, as a readout of membrane potential activity. Changes in neuron size that alter the activity of a neuron may therefore enable activity-dependent regulation mechanisms to compensate for size changes without requiring a separate mechanism to explicitly measure cell membrane area.

We investigated this hypothesis using single-compartment biophysical models of crustacean pacemaker neurons. The single-compartment assumption allowed us to model a dominant feature of neuron growth, the increase in the cell’s surface area and effective volume. This simplified model necessarily neglects the extension and branching of neurites and is therefore most relevant to electrotonically compact cells, such as CPG neurons, cardiac myocytes or muscle cells. However, our results and analyses are relevant to local regulation of conductances within compartments of spatially extended cells and can therefore inform studies of this more complex regulation problem.

Our model assumes a single sensor of calcium concentration to control expression of multiple ion channels simultaneously, a so called *master regulation* scheme. Previous work showed that this class of self-tuning models recapitulates experimental observations such a correlated variability in ion channel density and compensation for changes in circuit activity^17–22^.

**Figure 1:**
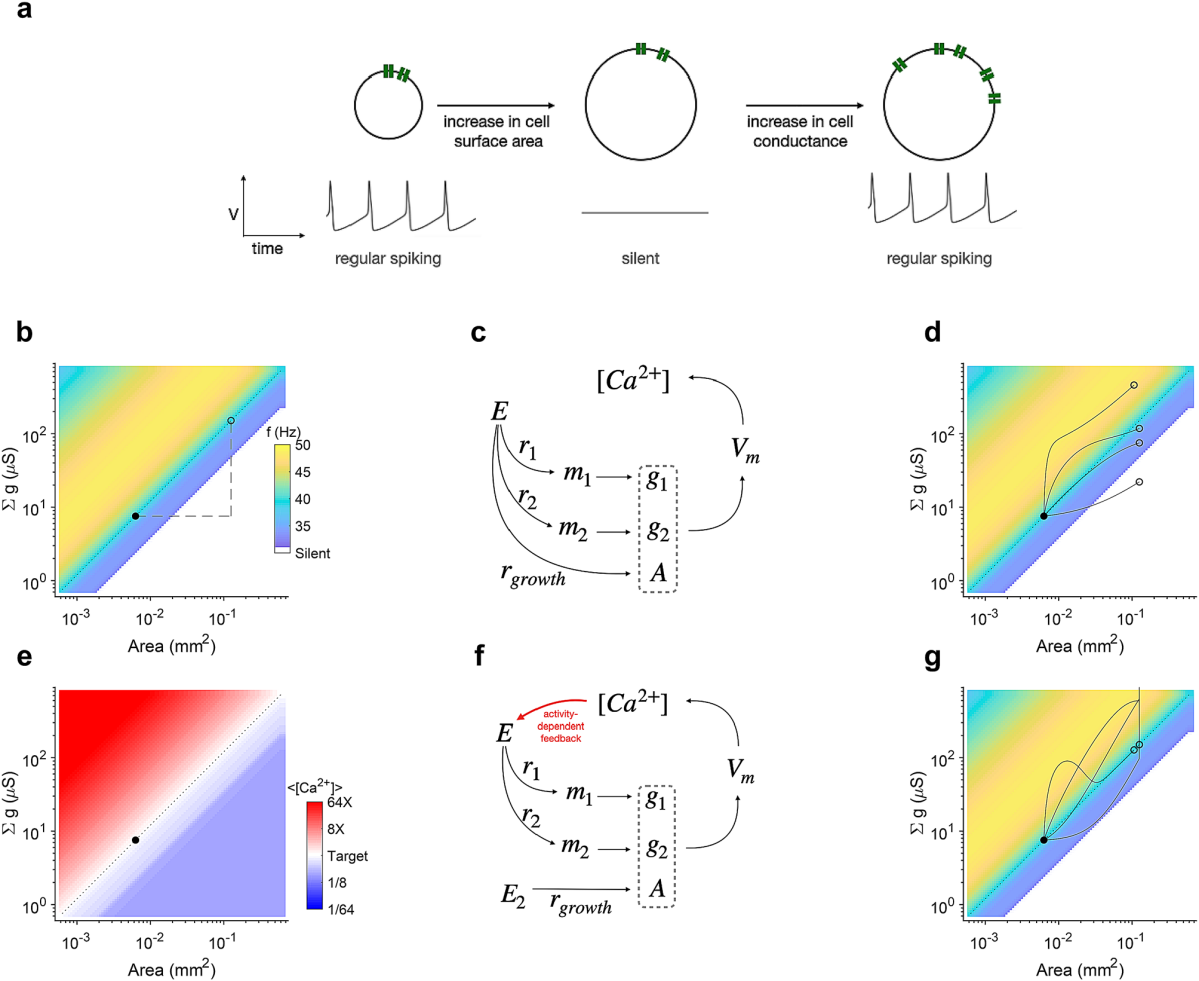
Activity-dependent regulation of conductances can robustly compensate for changes in cell size(**a**) A regularly spiking neuron can go silent as its cell surface area is increased. An appropriate increase in the conductance of all ion channels can restore the original firing behaviour. (**b**) Firing rate (colour code) of the neuron as a function of cell size and channel conductance. A neuron originally at the location indicated by the black dot must remain on the dotted line (the line of constant conductance density) as it grows to preserve function. Dashed lines indicate changes in (**a**). (**c**) A simple open loop scheme for co-regulating conductances and area. An effector up-regulates both channel conductances and membrane area with rates $r_1 \ldots r_{growth}$. (**d**) Open-loop co-regulation can increase both the area and the conductances, but remaining on the line of constant conductance density requires fine-tuning of growth rates. Four trajectories correspond to growth rates $[10^{-7}, 5\times 10^{-6}, 10^{-6}, 5\times 10^{-5}].$ (**e**) Average calcium levels in the cell are equal to the average calcium levels in the initial condition (the target) along the line of constant conductance density, suggesting that calcium can be used as a feedback signal to regulate conductances. (**f**) Modified regulatory scheme where calcium activity feeds back onto channel mRNA levels. (**g**) Feedback regulation is insensitive to growth rate, and in all four cases, the model settles onto the dashed line, and has the same voltage dynamics as in the initial condition.

In common with biological systems, these models exhibit robustness to some forms of perturbations in membrane conductances, but can be inherently sensitive to other perturbations like channel deletions^22^. This property is important, because in spite of widespread evidence for homeostatic compensation in the nervous system, there are clearly situations when such mechanisms can fail and potentially be the source of a pathology^23^. However, no study to date has shown what biologically relevant perturbations a master regulation scheme, such as that shown in Fig. 1f, is specifically suited to, nor analysed its specific vulnerabilities.

We show that a master regulation scheme based on a single sensor of calcium concentration is specifically suited to compensate size changes that would otherwise disrupt electrophysiological behaviour. By analysing how size changes perturb electrical properties, we construct a way to map perturbations that activity dependent channel regulation can compensate for as well as showing how and why certain perturbations cause regulation to fail. Our results suggest that biological regulation that is optimised for coping with ‘expected’ perturbations such as size changes will be vulnerable to other kinds of perturbations such as channel deletions.

## Results

### Activity-dependent regulation of conductances can robustly compensate for growth

We use a single-compartment neuron model (see “Methods and materials” section) with membrane potential $V_m$, and intracellular calcium concentration $[Ca^{2+}]$. We make the simplifying assumption that the effective volume accessible to calcium $\eta$ scales with the area of the cell *A*. This approximation, referred to as the *thin shell assumption*, is based on the following findings. First, it is known that calcium buffering and diffusion create microdomains of elevated intracellular calcium around calcium channels, suggesting that calcium influx does not lead to large increases in the calcium concentration across the entire volume of the neuron^24–26^. Second, an important method by which intracellular calcium affects neuron dynamics is by activating calcium-activated potassium (KCa) channels, which are known to localise close to calcium channels^27–29^. Third, fitting this model to experimental data revealed that the fast time courses of calcium-sensitive currents could only be reproduced by using a much smaller volume than the geometrical volume, consistent with the idea that the volume accessible to varying calcium is a thin shell within the membrane^30^.

How can a model neuron maintain its voltage dynamics during growth under these assumptions? We first recognise that neuron voltage dynamics depends only on conductance *densities*, rather than absolute values of conductances (see “Methods and materials” section). Therefore, maintaining conductance density during growth should be sufficient to preserve voltage dynamics Fig. 1a. Several strands of experimental evidence are consistent with the prediction that larger cells should have more channels, and should preserve conductance density.Input resistance decreases from juvenile to adult p1 muscles in lobster, suggesting that larger muscles had more channels^31^. Similarly, larger lumbrosacral alpha-motoneurons have lower input resistance^32^. Perturbations that increase cell size in *Aplysia* increase conductances of a specific channel type (A current), but preserve channel density^33^.

The principle of constant conductance density preserving voltage dynamics is illustrated in Fig. 1b: the firing rate of a model neuron is sensitive to surface area and to changes in the sum of all conductances (dashed lines), but remains constant along diagonal lines of constant conductance density (dotted line). Rightward movement in Fig. 1b, representing an increase in membrane area, must be compensated for by an appropriate increase in the conductance of all channels (Fig. 1b, dashed lines). What class of mechanism can steer a neuron along the line of constant conductance density?

One possibility is that a growth signal, *E*, synthesises new channel proteins and new membrane at some fixed set of rates. In the schematic shown in Fig. 1c, *E* controls the conductances $g_1$ and $g_2$ of ion channel populations by adjusting the mRNA levels $\mu _1$ and $\mu _2$ of those channels at transcription rates $r_1$ and $r_2$. *E* also increases the surface area of the cell by generating new membrane with rate $r_{growth}$. The set of parameters ${g_1, g_2, A}$ together determine the voltage dynamics of the cell, which in turn determine its calcium dynamics. This open-loop control mechanism can increase both the area of the cell and all conductances so that conductance densities and voltage dynamics are approximately conserved (Fig. 1d), but requires fine-tuning the growth rate $r_{growth}$. Mismatched growth rates can drive the neuron to parts of state space where the conductance density and spiking dynamics are substantially different (Fig. 1d). Moreover, any perturbation to the cell that affects its average activity cannot be compensated. Open loop regulation is therefore not a robust control mechanism.

We hypothesise that activity-dependent feedback is used to regulate conductances, because calcium levels are invariant along lines of constant conductance density, and therefore track voltage dynamics well (Fig. 1e). In this closed-loop control scheme (Fig. 1f), we assume a master regulator, *E*, that depends on the calcium concentrations in the cell^10,22^. Membrane area (growth) is assumed to be controlled independently (Fig. 1f). Feedback regulation can drive the neuron to the line of constant conductance density, and therefore the desired voltage dynamics, no matter what the growth rate is (Fig. 1g). Activity-dependent feedback regulation of channel conductances is thus a robust mechanism of compensating for growth.

### Necessary conditions for homeostatic feedback to be robust to size changes

**Figure 2:**
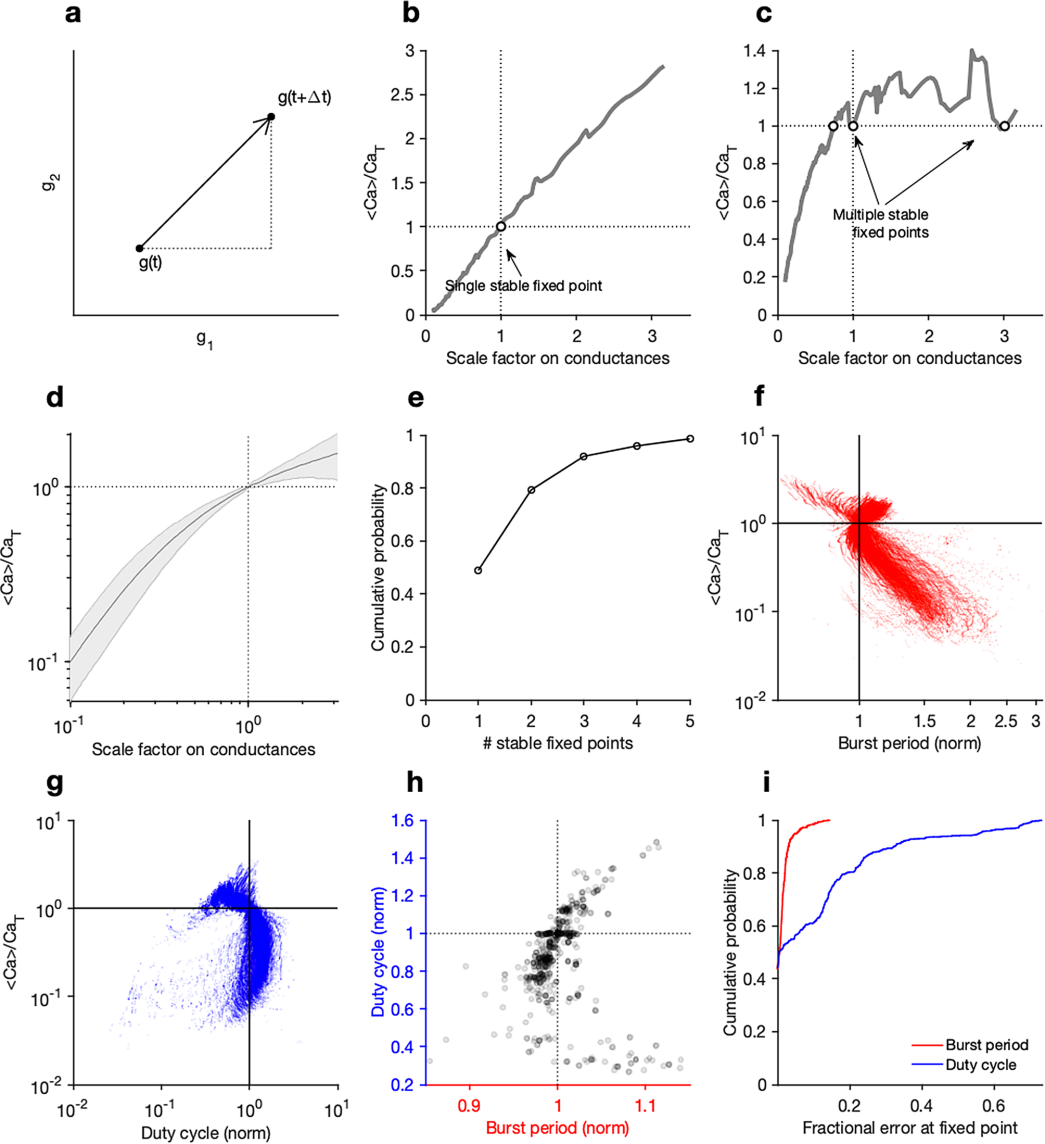
Changes in cell size can disrupt neuron dynamics, but can be compensated for by a mechanism that does not explicitly measure cell size. (**a**) A regulation system can move the neuron in the space of maximal conductance densities of its ion channels. Here, two populations are shown. Scaling all conductance densities together changes its mean intracellular calcium levels, leading either to (**b**) a single stable fixed point for the regulation system or (**c**) multiple stable fixed points. (**b**,**c**) show two different example neurons with different sets of maximal conductances. (**d**) Mean calcium levels, scaled by the target of the regulation system, as a function of scaling of all conductances for a population of bursting neuron models. Shading indicates standard error of mean. (**e**) Distribution of number of stable fixed points across population of bursting neurons (N = 635) . Almost all models have fewer than 5 stable fixed points. Average calcium levels vs. (**f**) burst period and (**g**) duty cycle of a population of bursting neurons, during scaling of conductances. Scaling conductances changes the burst period and the duty cycle, but it also changes the mean intracellular calcium concentration, suggesting that these perturbations can be compensated for by the regulation system. (**h**) Duty cycles and burst periods after compensation by regulation system. Most models are close to their target duty cycle and burst period (intersection of dotted lines). (**i**)Cumulative distributions of burst period and duty cycle for a population fo bursting neuron models after scale perturbation of ion channels and compensation at fixed points of regulatory system.

In the previous section, we showed that activity-dependent feedback regulation can compensate for growth and keep neurons along the line of constant conductance density. Instantaneous changes in cell size corresponded to horizontal motion along the space shown in Fig. 1a, which resulted in a coordinated scaling of all conductance densities. For feedback coregulation to be robust to size changes, it must therefore be robust to perturbations of this type. To determine the necessary conditions for this to occur, we first observe that even though this regulatory system can control many different ion channel types, all motion is constrained to lines in the space of conductance densities. To show this, consider feedback regulation controlling two conductances, $g_1$ and $g_2$. After some small time $\Delta t$, regulation may change these conductances, as shown in the schematic in Fig. 2a.The direction of this change $\theta$ is given by

$$ \begin{aligned}   \tan \theta  =\,  & \frac{{g_{2} (t + \Delta t) - g_{2} (t)}}{{g_{1} (t + \Delta t) - g_{1} (t)}} \\     =\,  & \frac{{\dot{g}_{2} \Delta t + g_{2} (t) - g_{2} (t)}}{{\dot{g}_{1} \Delta t + g_{1} (t) - g_{1} (t)}} \\     \approx  \,& \frac{{\tau _{{\mu _{1} }} }}{{\tau _{{\mu _{2} }} }} \\  \end{aligned}  $$

This is true for all pairs of conductances when more than two conductances are coregulated. Therefore, the only direction this regulatory scheme can move in is either parallel or anti-parallel to the direction caused by growth in the space of conductance densities. The problem of determining if regulation can compensate for size changes is thus reduced to the problem of finding stable fixed points along the ray from the origin to the point in conductance density space the neuron is located at. The fixed points of the regulatory system along this ray are determined entirely by how the calcium concentration changes with motion along this ray (Fig. 2b,c). Some models can exhibit nearly monotonic increases of calcium with distance along the ray (as in Fig. 2b), and other models can manifest multiple stable fixed points (as in Fig. 2c).

To determine if calcium concentrations are typically non-monotonic with motion along the growth ray, we generated a database of 635 bursting neurons with similar burst periods and duty cycles (see “Methods and materials” section), randomly sampled from the 8-dimensional space of conductances. We measured how calcium varied with distance along this ray in all models (Fig. 2d). Over 80 percent of randomly sampled models had 1 or 2 stable fixed points (Fig. 2e), suggesting that numerous stable fixed points were not generically observed. Visualising how burst period (Fig. 2f) and duty cycle (Fig. 2g) varied with the calcium concentration revealed narrowing of both distributions at the target calcium level, suggesting that the one-dimensional readout of the average calcium activity could act as a good proxy for bursting voltage dynamics.

Even if multiple stable fixed points are present for a particular set of conductances, it does not necessarily mean that homeostasis cannot be robust to size perturbations, since more than one fixed point can manifest similar voltage dynamics. To determine how close the voltage dynamics of the neuron at stable fixed points were to the desired voltage dynamics, we plotted duty cycle and the burst period at all detected stable fixed points in all neurons, normalised by the duty cycle and burst period of the desired voltage dynamics (Fig. 2h).Around 80% of all voltage dynamics at stable fixed points had burst periods and duty cycles within 20% of the desired voltage dynamics (Fig. 2i), suggesting that conductance sets that can be regulated in a size-robust manner are abundant in the space of all conductances. Single-sensor co-regulation can thus reliably compensate for size changes in a large fraction of neuron models.

### Robustness to size change perturbations coexists with sensitivity to some channel-specific perturbations

We have shown that homeostatic regulation can compensate for scale perturbations such as growth, but it remains unclear why it can sometimes fail for other, seemingly smaller perturbations such as when channels are knocked down or over-expressed^12,19,22,34^. Acute sensitivity to perturbations can vary across neuron types. Furthermore, homeostatic regulation can compensate for a perturbation, or produces pathological responses in some perturbations that might be otherwise benign^22^.

In our framework, size changes are equivalent to a perturbation in which all maximal conductances are scaled together. An arbitrary perturbation in a neuron with *N* ion channel types is a *N*-dimensional vector, this will typically be incongruent with a scaling. This suggests that homeostatic regulation may be good at compensating for perturbations in some directions, but not others.

To quantitatively test this prediction, we analysed response to perturbations, including size changes, in an 8-conductance neuron model that can exhibit a rich variety of voltage dynamics^12,35^, including tonic spiking and bursting.

**Figure 3:**
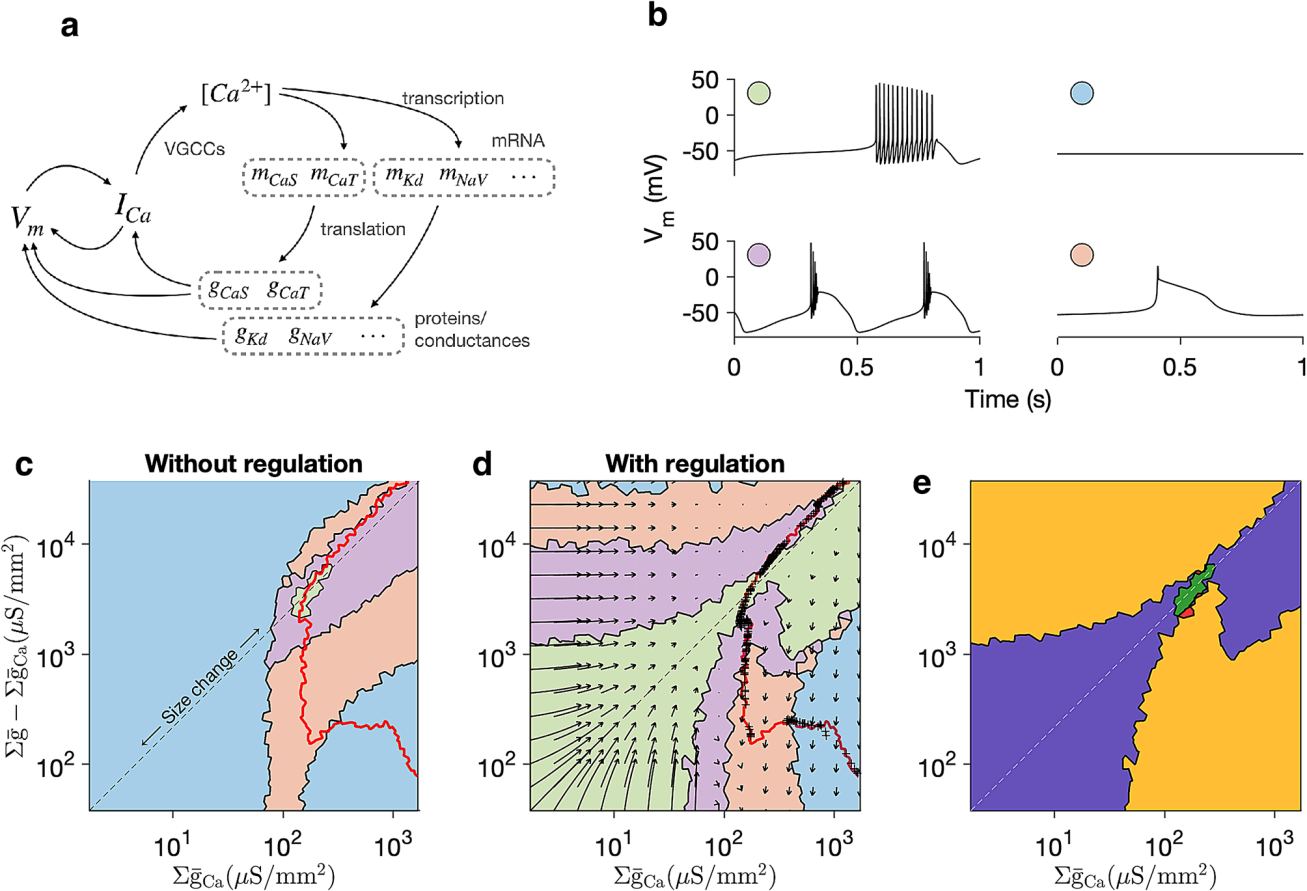
Ratio-preserving regulation rules confer robustness to size changes, at the cost of sensitivity to orthogonal perturbations. (**a**) Homeostatic feedback loop segregated into variables that directly affect intracellular calcium and those that do not. (**b**) In this neuron model, four classes of neuron dynamics emerge as conductance densities are varied: canonical bursting (green), silent (blue), non-canonical bursting (purple) and one-spike bursting (pink). (**c**) Distribution of these dynamical classes across space of conductance densities. The diagonal corresponds to changes in cell size. The red line joins points in this space where the average intracellular calcium is equal to the average of the reference model. Along the red line, the regulation mechanism, no matter what its kinetic parameters, will be inactive. (**d**) Basins of attraction of different dynamical states with homeostatic regulation. Cells perturbed along the diagonal (corresponding to a change in size) return to the reference point in conductance space. Perturbations that are further from the diagonal lead to other states. (**e**) Segmentation of space based on response to perturbation and compensation: robust to perturbation, compensation restorative (green); sensitive to perturbation, compensation restorative (purple); sensitive to perturbation, compensation pathological (yellow); robust to perturbation, compensation pathological (red).

Understanding the dependence of this homeostatic feedback mechanism in an 8-dimensional space is challenging. Due to the dependence of the feedback mechanism on intracellular calcium, we reasoned that the 8 ion channel types in this model could be grouped into two sets based on whether they directly affected intracellular calcium or not (Fig. 3a). Using a simple classification scheme to group neuron behaviours into one of four non-overlapping classes (Fig. 3b), we then projected the 8-dimensional space of conductance densities onto a two-dimensional plane (Fig. 3c). Regular bursting dynamics with burst periods and duty cycle within 10% of the target neuron were classified as “canonical” (green). Bursting dynamics outside this range were classified as “other bursting” (purple). The other two states were a silent state (blue) and a tonically spiking state (pink).

We then calculated a level set of calcium where average intracellular calcium levels were equal to that of the target model (Fig. 3c, red line). This line passed through all four states, suggesting that many different states could result from homeostatic compensation, even for neuron models with different parameters.

To determine which perturbations could be recovered from, we numerically calculated the basins of attraction of these four dynamical states by initialising models at different points in the plane (Fig. 3d). In this example, all perturbations that move the conductance densities of the neuron within the green basin can be compensated from, because homeostatic regulation drives the conductances back to the target state, and the neuron eventually generates its original voltage dynamics. There was a total overlap between the basin of attraction of the target state and the diagonal, suggesting that all size change perturbations can be compensatedperfectly. Strikingly, the basin of attraction of the target state was relatively broader close to the origin and relatively narrower at the reference state. This indicates that highly disorganised expression can be compensatedat low absolute levels of ion channel densities, as would be typical in a developing neuron.

Robustness to perturbations along the diagonal (corresponding to changes in cell size) coexisted with sensitivity to off-diagonal perturbations. Mapping the flow field in this plane (Fig. 3d) reveals that flows close to the diagonal are restorative and drive the neuron to the original set of conductance densities, while flows far from the diagonal can drive the neuron to homeostatic targets far from the reference model. Thus, perturbations in a direction orthogonal to the diagonal can lead to a very different steady-state outcome, resulting in pathological compensation.

We constructed a map of the sensitivity of feedback regulation to perturbations (Fig. 3e). Only a small region corresponded to acute robustness to perturbation (Fig. 3e green). A much larger region, along the diagonal, corresponded to sensitivity to perturbation but where homeostasis could compensate for the perturbation (Fig. 3e purple). Intriguingly, regions where the neuron was acutely robust to perturbations existed close to the reference model, where compensation was pathological (Fig. 3e red). These regions, though close to the reference model, did not lie on the diagonal. This suggests that ratio-preserving regulation rules, which may have evolved biologically to confer robustness to size changes, are vulnerable to perturbations in the expression of specific channel types.

### Predicting failure of homeostatic compensation

In the previous sections, we showed that that the calcium level sets correspond not only to the desired physiological bursting dynamics, but also other bursting dynamics, tonic spiking, and silence. The existence of these regions of parameter space are necessary, but not sufficient, for a pathological homeostatic outcome because the error of the feedback signal in these regions is zero.

To find sufficient conditions for failure of a calcium-dependent feedback mechanism, we focussed on the region in parameter space where calcium level sets coincided with silence. While previous work had also suggested that homeostatic compensation to perturbations such as channel deletions could render neurons silent, it remains unclear, mechanistically, why some perturbations can be compensated for, and why some render the neuron silent, and cannot be recovered from. Periods of silence have been observed during ‘crashes’ when experimental perturbations exceed the permissive range, and are typically beyond neurons’ ability to compensate^36–38^.

**Figure 4:**
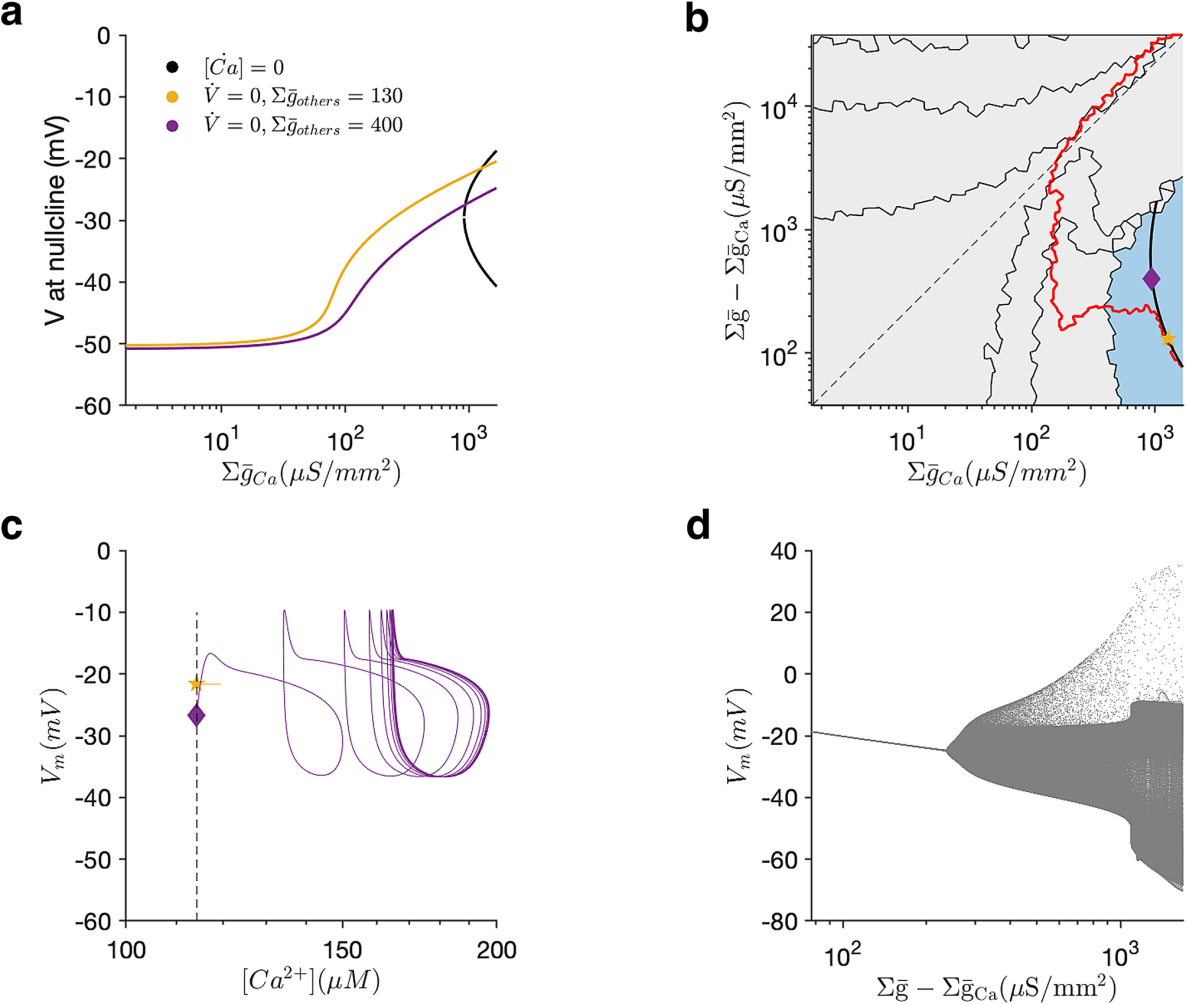
Analytical prediction of when perturbations can silence the neuron. (**a**) Values of membrane potential corresponding to zeros in the calcium or voltage ODE. The intersection of coloured and black curves corresponds to fixed points in the neuron dynamics. (**b**) Segmentation of conductance density space highlighting the basin of attraction of the silent state (blue). Red line is the calcium level set computed by numerically integrating the model. Black line is the analytical prediction of the location of silent solutions. Pentagram (yellow) and diamond (purple) indicate two locations along the analytically predicted silent state that we shall examine more closely. (**c**) Dynamics of a neuron model without homeostasis when initialised at these two points. The state indicated by the pentagram (yellow) is stable, but the state indicated by the diamond (purple) is not. Dashed line indicates target calcium of homeostatic regulation. (**d**) Membrane potential of the neuron along the analytical solution for the silent state, parameterised by the Y-axis in (**b**). At a critical point, the quiescent state is destabilised, corresponding to the point where the analytical solution and the calcium level set diverge in (**b**).

We hypothesised that homeostatic rules can trap neurons in silent states when the membrane potential is sufficiently depolarised so that a constant influx of calcium through calcium channels occurs due to window currents. We assumed that the silent state corresponds to quenched dynamics in both voltage and calcium. Because the calcium level is constrained to be the same as that of the reference model (the calcium target), we can solve for the fixed points of Eq. (3) to obtain the voltages at which the calcium dynamics are quenched. We note that voltages at which these fixed points occur do not depend on channels that are not calcium channels, since they do not affect calcium dynamics. Fixed points in the calcium ODE exist only for large values of the calcium channels conductance density (Fig. 4a), suggesting that silent states that cannot be recovered from are impossible below a critical calcium channel conductance density.

Similarly, we can solve for fixed points in the voltage ODE (Eq. (2)). The intersection of these two sets of curves corresponds to fixed points in both the voltage and calcium dynamics (Fig. 4a). Plotting the location of points in parameter space where both fixed points overlap reveals a smooth line (black) that partially overlaps with the calcium level set (red), and is entirely contained in the numerically computed basin of stability of the silent state (Fig. 4b).

Why does the analytically calculated set of silent states contain a branch that does not overlap with the numerically computed calcium level set? One possibility is that while all points in the analytical set are indeed silent, their stability properties change along the curve. The voltage fixed points are always unstable, and the upper branch of the calcium fixed points are always stable, suggesting that the overall stability of the silent state may depend on which dynamics dominates as a function of position along the set. To test this, we examined two points along the analytical set, one where it coincided with the numerically measured calcium level set (Fig. 4b, yellow star), and one on the branch that diverged from the numerically measured calcium level set (Fig. 4b, purple diamond). While a neuron initialised at the yellow star remained quiescent (Fig. 4c), a neuron initialised at the purple diamond spontaneously left the silent state, and settled on a periodic sub-threshold orbit (Fig. 4c). Plotting the voltage as the parameters of the neuron model are varied along the analytically calculated set reveal that the neuron switches from silence to sub-threshold oscillations to spiking (Fig. 4d), which is caused not by the regulatory mechanism but by a destabilisation of the fixed point in the intrinsic voltage and calcium dynamics of the neuron.

Together, these results show that simple, calcium-dependent channel regulation mechanisms can be inherently sensitive to channel deletions and produce pathological compensation, even for perturbations that may not affect neural behaviour acutely.

### Robustness to scale perturbations persists across projections and neuron models

Up to this point, we have analysed the effect of perturbations using a specific projection of the high dimensional space of conductances. The dimension of the full space of conductance densities is equal to the number of distinct ion channel populations *N*, and the projections shown in the preceding sections do not capture the full space. Similarly, the full level set of calcium is also high dimensional, since it exists in the full *N*-dimensional space, and appears as lines in these projections only because intersections with the projection plane are plotted. The projection chosen in the preceding sections emphasised the distinct contribution of calcium currents, leading to the question if the general features seen hold true for other projections.

**Figure 5:**
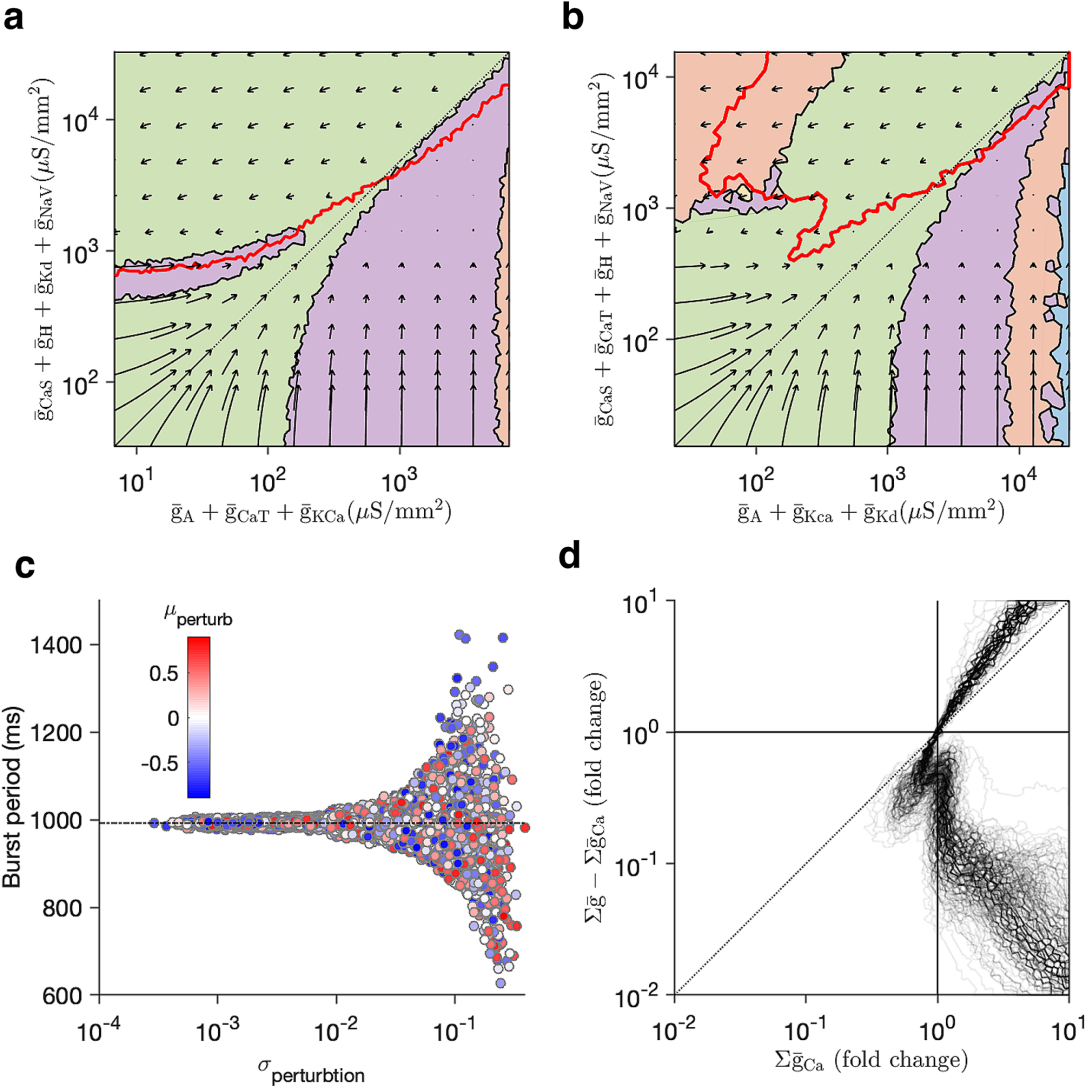
Robustness to scale perturbations persists across projections and neuron models (**a**,**b**) Basins of attractions of target dynamical state (the canonical bursting state, green) and calcium level sets (red lines) for two additional projections. (**a**) Projection onto arbitrary subsets of conductance densities. (**b**) Projection onto conductance densities of outward (X) and inward (Y) currents. In both (**a**,**b**) the diagonal is contained in the basin of attraction of the target state (green), and the diagonal intersects the calcium level set only once. (**c**) Burst period after homeostatic compensation to perturbations parameterised by mean and standard deviation. (**d**) calcium level sets for a database of 350 neuron models that share similar voltage dynamics but differ in their conductance parameters. Despite large variation in conductance values and ratios of maximal conductances, in almost all models, the level sets intersect with the diagonal only once.

We repeated the perturbation analysis for two additional perturbations (Fig. 5a,b). No matter what projection is chosen, the diagonal always corresponds to a change in size, since along that line all conductance densities are scaled together. For both additional projections, we found that the diagonal is entirely contained in the basin of attraction of the canonical state (Fig. 5a,b, green zone) and that the calcium level set intersects with the diagonal exactly once. Taken together, these results suggest that scale perturbations (size changes) can typically be compensated for by homeostasis, but off-diagonal perturbations (e.g., channel deletions, pharmacological manipulations) may not be.

A generic perturbation in the conductance densities of ion channels in a neuron is high-dimensional, and does not occur on a plane. What effect does a typical perturbation have on the homeostatic system, and what features of the perturbation determine if compensation is restorative or pathological? We parametrised random perturbations in the full dimension space by their mean and variance (relative to the conductance densities in the original model, see “Methods and materials” section), and measured the burst period of neuron models after recovery from perturbation (Fig. 5c). Consistent with our two-dimensional perturbation analysis, the ability of the homeostatic mechanism to recover from perturbations depended not on the mean value, but on the variance, suggesting that homeostasis could be simultaneously robust to large size changes (low-variance, correlated changes) and sensitive to ratio-disrupting perturbations.

Theoretical and experimental work has shown that many different sets of maximal conductances can lead to similar voltage dynamics^35,39–45^. Are all models that share a similar dynamics equally robust to changes in size and off-diagonal perturbations? Picking a regularly bursting neuron with a well-defined burst period and duty cycle, we searched the 8-dimensional space of conductance densities to find  350 sets of maximal conductance densities that displayed similar dynamics, though the variation of individual conductance densities and ratios of conductance densities was significant. For every model, we measured the average intracellular calcium at baseline, and then computed a level set of calcium for every model where the set of calcium was the same as in baseline. Despite the large variation in individual parameters, all calcium level sets were strikingly similar, and almost all level sets intersected with the diagonal exactly once (Fig. 5d). The similarity of calcium level sets across neuron models suggests that different models with diverse maximal conductance densities arerobust to changes in size, and can behave similarly to perturbations in their ion channels.

## Discussion

Cellular growth is typically ignored as a potential source of disruption to physiological properties, yet all cellular systems require cell volume and area to be regulated in concert with cellular signalling. Electrophysiological properties of cells are particularly sensitive to cell capacitance and channel density. As a consequence, growth, as well as variation in cell size across a population, would prevent the emergence and maintenance of appropriate physiological properties without some form of feedback. We show that a very simple and plausible form of feedback that jointly regulates multiple conductances is specifically suited to maintaining electrophysiological properties despite changes in cell size. Furthermore, we analyse the specific vulnerabilities of this regulation scheme, revealing its sensitivity to targeted perturbations in specific ionic conductances.

Our approach also provided a very concrete illustration of why it is difficult to understand the function of biological systems by deleting or perturbing components individually or acutely. By mapping the behaviour of neural activity to channel perturbations both in the presence and absence of homeostatic feedback (Fig. 3c,d), we see that the relationships between channel perturbations and overall behaviour are fundamentally different. This is expected, but it is important to note that many studies use perturbations that are either acute or chronic. As a consequence, the intervening effect of any compensation (pathological or otherwise) cannot be accounted for. The stark difference between open loop (acute) and closed loop (chronic) in our work prompts caution for any study that attempts to understand the sensitivity of a system to perturbation, whether experimental or in simulation.

### Calcium as a proxy for cell activity

For activity-dependent homeostatic regulation to work, a neuron must be able to measure the deviation of its own activity from some target. A common hypothesis, that we have adopted in this paper, is that neurons use intracellular calcium concentrations as a proxy for activity^10,11,46–49^. Because baseline levels of intracellular calcium concentration are very low compared to extracellular levels, depolarisation of the membrane that opens calcium channels can lead to a transient calcium influx. Calcium channels therefore can play an important role in directly mapping the voltage dynamics of a neuron onto its intracellular calcium levels. Intriguingly, calcium channels are expressed early in development, suggesting that this part of the feedback loop precedes regulation and expression of ion channels needed for the neuron’s target behaviour^50–54^. Finally,the function of ion channels is deeply intertwined with the maintenance of ion gradients across the cell membrane, and therefore the regulation of osmotic pressure. Therefore, using ion concentration, especially the concentration of a tightly controlled ion like calcium, to act as a proxy for cell activity and cell size emerges naturally.

However, our results suggest that having too many or too large a fraction of calcium channels can lead to undesirable outcomes, where calcium window currents can fool homeostatic regulation into perpetuating silent states (Fig. 4). Intriguingly, a number of studies of calcium-dependent regulation show that calcium influx through specific channels is required for homeostatic responses, which may make homeostatic regulation more robust biologically^16,55^. Developing and growing neurons likely switch regulation rules during their lifetime, using one rule to express channels needed for function and another to maintain ionic conductances within acceptable limits^56^, suggesting that neurons may avoid some forms of pathological compensation by switching regulation rules based on developmental context.

### Consequences of modeling calcium concentration using a single variable

One of the consequences of the simple single-compartment model we have used in this paper is that we use a single variable to describe (a) the calcium concentration in the thin shell that affects the gating of KCa channels, (b) the calcium concentration in the bulk of the soma that likely determines rates of translation, insertion or degradation of ion channel proteins (Eq. (5)), and (c) the calcium concentration in the nucleus, that modulates transcription rates (Eq. (4)). In real neurons, these three calcium concentrations can likely be very different^57,58^, and can have different scaling properties. For example, while the thin-shell calcium concentration can scale with the surface area of the cell, the bulk calcium concentration may scale with the volume of the cell. While this model lacks the details to tease these disparate effects apart, it is useful to consider the biophysical models that are consistent with the assumptions we made to simplify our analysis.

The discovery of protein synthesis in neuronal compartments far from the soma, including in dendrites, suggests that local activity-dependent mechanisms can regulate local protein levels^59–61^. Such mechanisms likely depend on concentration of calcium in some small neighbourhood, rather than the calcium concentration in the soma, suggesting that a one-compartment model can mimic some features of local, activity-dependent protein synthesis and degradation^62^. Another possibility is that detachment of granules of mRNA at a particular location^63^, or the insertion or removal of ion channels into the membrane, can depend on the local calcium concentration. In summary, local activity-dependent regulation mechanisms can be approximated by the model used here, and are an attractive formalism since they do not require co-ordination of sensors and regulators across the neuron.

### Complex morphologies present further challenges for homeostatic regulation

We examined how neurons could use homeostatic mechanisms to regulate their ion channel conductances to preserve intrinsic dynamics as their size changed in a single compartment model. A single compartment model neglects many of the complexities in real neurons since the neuron is described by a single membrane potential and a single value of intracellular calcium and is assumed to have no internal structural heterogeneity. Studies have shown that organelles typically scale with cell size^64^, and their size may be co-regulated with lipid biosynthesis^65^. Our simplifying assumptions prevent us from studying these interactions. While single compartment models are clearly only a coarse approximation of real neurons, recent work has suggested that some neurons are surprisingly electronically compact despite their large size and spatially complex morphology^66,67^.

Other cell types with complex morphologies that change during growth (e.g., motor neurons controlling muscles in the extremities) face several additional regulatory challenges. First, their dynamics are no longer described by a single membrane potential since they are not electronically compact. Second, channel expression is spatially regulated: for example, sodium channels are expressed at a higher density on the axon^68^. Third, intracellular calcium levels are not spatially uniform^69^, and can vary substantially along the length of thin processes^24^. Finally, since all mRNA ultimately originates from the cell body, mRNA and ribosomes and other translation machinery, or entire ion channels, must be physically transported from the nucleus to parts of the cell where they are needed^63,70,71^, leading to other regulatory problems with the transport of cargo^63,72^. We hypothesise that the distributed regulation problem may be understood by breaking spatially extended cells into local compartments where activity signals and the pool of available channels and expression machinery may be assumed to be well-mixed. Under such assumptions the analysis we developed here could provide a starting point for a model of local, distributed regulation. Further work, including our own ongoing work^73^, can connect such local regulation to the global regulation at the level an entire, spatially extended cell.

### Stochastic effects can arise from low copy numbers

Following earlier work, we modelled the gating of ion channels as a deterministic process^74^. Even though the gating of an individual ion channel is a stochastic process, this deterministic model approximates well a large number of ion channels, since their individual fluctuations average out^75^. In most neurons and animal cells the total membrane conductance corresponds to channel counts typically in excess of $10^3$ (e.g. small *Drosophila* central neurons with input resistance $R_m \approx 500$ M$\Omega$, *shaker* channel open conductance $g_{open} \approx 10$ pS, and open probability at resting potential $p_{open} <10\%$).

However, when neurons are sufficiently small, a neuron, or sub-compartment of a neuron may have fewer than 100 channels^76^. In this limit, the stochastic gating of ion channels becomes substantial, and has been shown to qualitatively change the behaviour of neurons^77,78^. Another source of stochasticity is in the regulation mechanism since mRNA copy numbers are typically low^70^. It is not clear whether homeostatic mechanisms continue to function in these extreme limits, and there is formal theory to show that low copy numbers present regulation problems that cannot be circumvented^79^. A requirement for regulation may therefore place a lower limit on channel expression density in addition to requirements imposed by reliable signalling^80^.

However, growth tends to move the cell away from this limit into a regime where our modelling assumptions are more relevant. Furthermore, the results in Fig. 3e illustrate that coordinated channel expression can emerge from a highly disorganised state where conductance ratios are undefined. Such a transition from a disorganised initial state to a state with well-defined physiological properties may be relevant during development, when cell identity is established and the expression of key genes is low^81^.

### Non-neuronal cells

Our results suggest that cells that generate stereotyped voltage dynamics despite potential changes in size can use a single sensor, for instance intracellular calcium, to regulate abundances of several different protein types that contribute to the voltage dynamics. Neurons are the archetypal electrically active cells, and can realise this negative feedback loop using calcium-dependent transcription, translation, or channel insertion, voltage-gated channels, and calcium channels. Stereotyped electrical activity is widespread in many different cell types, including bacteria^82,83^, pancreatic $\beta$-cells^84^ and cardiac cells^85^. Bursting oscillations play an integral role in insulin secretion by pancreatic $\beta$-cells^84^, and these cells can compensate for genetic deletions of a critical channel K(ATP) channel population by over-expressing other potassium channels^86^ in the mouse, but not in humans. Recent work suggests that these cells can use intracellular calcium as a sensor of voltage dynamics in a negative feedback loop to regulate activity^87^. Thus, many other cell types may share common features of homeostatic regulation with neurons such as robustness to size changes and sensitivity to some perturbations^88^.

### Other mechanisms of compensation for growth

In this study, we analysed how neurons can regulate the densities of ion channel populations as they grow. In addition to this, neurons can regulate a number of other properties during growth. The manner in which a neuron grows can have a critical role in shaping neuronal function. In *isometric* growth, the length and diameter of a neuron or neuron component increase by the same factor, which increases input conductance by the square of the growth factor, and changes the passive properties of the neuron. In *iso-electrotonic* growth, the diameters of neuronal processes increase as the square of their increase in length, which increases input conductance by the cube of the growth factor, but leaves passive properties of the neuron unchanged^89^. Lateral giant neurons in the crayfish grow isometrically, and become progressively less sensitive to phasic components of inputs, since high-frequency signals are attenuated to a greater extent^90,91^. Retzius neurons in the leech can compensate for an increase in size by increasing the membrane resistance of the dendrites^1^.

Compensation of size change in a population of neurons can also be achieved via the interaction of regulatory mechanisms at the circuit level. In pyramidal cells, as dendrites grow, pre-existing synaptic sites become physically or electrically more distant to the soma^92,93^. Muscle cells in the crayfish neuromuscular junction maintain a constant level ofdepolarisation, despite a 50-fold increase in their size, by a regulated increase in the presynaptic release and quantal size^92,94,95^. Circuits early in development can express adult-like rhythmic activity, but their activity can be continuously inhibited by descending cells^4,96,97^. Neuromodulators and co-transmitters^98,99^ can be sequentially released onto neurons during development, which means that the neuromodulatory context a neuron exists in may depend on its size. Homeostatic mechanisms like synaptic scaling that can compensate for changing levels of input can also help a neuron compensate for changes in input resistance that may occur during growth^15,100^.

### A hypothesis for conserved regulatory motifs across cell types

In neurons, ion channel expression needs to be regulated to maintain excitability across variations in cell size and during growth. We have shown that a robust way of achieving appropriate regulation is to use a common co-regulator that is coupled to ion concentration, since the direction of compensatory action is congruent to the direction of perturbation introduced by growth. However, the evolution of ion channels and ion transport predates the evolution of electrical signalling and excitability^88,101^. Ultimately, ion transport evolved as a mechanism for controlling cell volume by counteracting severe osmotic gradients that arise when proteins and macromolecules are packed into water-permeable cell membranes.This means that ion channel expression is intimately linked to cell size. Because this is a theoretical study, our findings cannot be taken as evidence of the existence of size-compensating regulatory mechanisms. Instead, our aim is to make connections that may not be obvious and to stimulate future experimental work. We speculate that analogous master regulation motifs that couple multiple ion transport-related components to ion concentrations exist across many cell types. We reiterate that these speculative ideas have not been experimentally tested, but we believe that our analysis of a simple regulatory model might provide incentive to test the hypotheses that regulation motifs are conserved and that ion flux might be used as an indirect readout of cell size in such regulatory motifs, should they be found.

Our work provides a mechanistic hypothesis for how and why regulatory systems can fail to compensate for deletions of ion channel types: it is reasonable to expect homeostatic mechanisms to have been evolutionarily tuned to make physiology robust to cell size and variations in passive properties because these change during development. The mechanism we put forward is a parsimonious way to achieve this that is also compatible with correlated variability in channel expression^21,102^. A mechanism that is tuned specifically to deal with these perturbations is necessarily vulnerable to channel deletions.

## Methods and materials

### Neuron model

We used a simple single-compartment conductance-based neuronal model with a single membrane potential *V*, that evolves according to

1 $$\begin{aligned} C{\dot{V}}=\sum _{i}{\bar{g}}_{i}m_{i}^{p}h_{i}^{q}(V-E_{i})A \end{aligned}$$

where *C* is the membrane capacitance of the cell, ${g}_i$ is the maximal conductance, $E_i$ the reversal potential, and *m* and *h* are activation and inactivation variables of ion channel population *i*. We note that we can divide this equation by the area to obtain

2 $$\begin{aligned} C_{m}{\dot{V}}=\sum _{i}{\bar{g}}_{i}m_{i}^{p}h_{i}^{q}(V-E_{i}) \end{aligned}$$

where $C_m$ is the specific membrane capacitance of the cell. We note that these dynamics do not depend on geometrical properties like the area *A* of the cell or the thin-shell volume $\eta$. In Figs. 2, 3 and 4, we used an eight-conductance model with these channels: *A*, *CaS*, *CaT*, *H*, *Kd*, *Leak*, *NaV* and *KCa*. Gating functions for all channels were identical to^35^.

Another important state variable of the neuron is the intracellular calcium concentration $[Ca^{2+}]_{in}$. In neurons, calcium enters the cell through ligand-gated- and voltage-gated calcium-permeable channels, increasing the cytosolic concentration^24^. Extensive intracellular stores can buffer this calcium influx, and can also act as both a source and a sink of calcium^103^. Following earlier work that modelled calcium dynamics in neurons^30,58^, we assumed that intracellular calcium concentration evolves according to:

3 $$\begin{aligned} \frac{d[Ca^{2+}]}{dt}=-\frac{\phi i_{Ca}A}{2F\eta }-\frac{[Ca^{2+}]-[Ca^{2+}]_{\infty }}{\tau _{Ca}} \end{aligned}$$

where $\phi$ is a dimensionless parameter, $i_{Ca}$ is the total calcium current density, *A* is the surface area of the cell, $\eta$ is the volume of the cell available for calcium influx, $\tau _{Ca}$ is the buffering timescale for calcium, $[Ca^{2+}]_{\infty }$ is the resting intracellular calcium concentration and *F* is the Faraday constant.

The first term represents calcium influx through voltage-gated calcium channels and the second term represents buffering by intracellular stores, diffusion, and extrusion out of the neuron. The size of the neuron directly affects this equation as both the area of the cell *A* and the volume relevant to calcium influx $\eta$ appear in the first term. Furthermore, the lumped timescale of calcium buffering $\tau _{Ca}$ also depends on the size of the neuron, but we do not consider its effect on size change in this analysis.

The reference model in Fig. 3 had the following maximal conductances (in units of µS/mm$^2$): $g_A$ = 379, $g_{CaS}$ = 165, $g_{CaT}$ = 2.35,$g_H$ = 0.72, $g_{KCa}$ =297, $g_{Kd}$ = 1713, $g_{Leak}$= 0.46 and $g_{NaV}$ = 1370. Following previous work^22^, regulation time constants ($\tau _m$) were obtained by inverting all maximal conductances and multiplying a constant (5000 ms).

### Regulation model

We used a simple model of homeostatic regulation in which transcription and translation depend on the deviation of time-averaged intracellular calcium from some target^22^. The concentration of mRNA that encodes channel protein *i* is given by

4 $$\begin{aligned} \tau _{\mu _{i}}\dot{\mu _i} = Ca_{target} - [Ca^{2+}] \end{aligned}$$

where $\tau _{\mu _{i}}$ is the timescale of transcription and $Ca_{target}$ is the average intracellular calcium concentration during target dynamics. Transcription and degradation affect the conductance levels of each channel type according to

5 $$\begin{aligned} \tau _{g}\dot{g_i} = \mu _i - g_i \end{aligned}$$

For a given model with some set of conductance densities ${\bar{g}_{i}}$, we picked regulation parameters so that

6 $$\begin{aligned} \frac{g_{j}}{g_{i}}=\frac{\tau _{\mu _{i}}}{\tau _{\mu _{j}}} \end{aligned}$$

for all pairs *i*, *j*. Following earlier work^22^, we set $\tau _g$ to be 5 s for all models. While this timescale is probably much longer in reality, we found that this value, which is larger than the slowest timescale of the voltage dynamics, was sufficiently large, and sped up simulations. With these parameters, this homeostatic control scheme could maintain baseline dynamics.

### Growth model

In Fig. 1, we dynamically change the size of the neuron using either a linear growth model. The area and the thin-shell volume of the cell change at a constant linear rate (${\dot{A}} = k, {\dot{\eta }} = k$).

### Analytical calculation of silent state

A neuron that is silent despite homeostatic regulation must satisfy the constraint that its average intracellular calcium levels are equal to the homeostatic target, $<[Ca^{2+}]>=Ca_{target}$. While in principle this condition can be met while having small oscillations in both *V* and $[Ca^{2+}]$, we make the simplifying assumption here that the silent state corresponds to fixed points in the calcium and voltage dynamics:

7 $$\begin{aligned} \frac{d[Ca^{2+}]}{dt}=0=-\frac{\phi i_{Ca}A}{2F\eta }-\frac{Ca_{target}-[Ca^{2+}]_{\infty }}{\tau _{Ca}}. \end{aligned}$$

This allows us to solve for $i_{Ca}$. While there is no closed form expression for *V* at which this equation is satisfied, we can easily calculate it using

8 $$\begin{aligned} i_{Ca}={\bar{g}}_{CaS}m_{CaS}^{3}h_{CaS}(V_{Ca}-E_{Ca}) +{\bar{g}}_{CaT}m_{CaT}^{3}h_{CaT}(V_{Ca}-E_{Ca}), \end{aligned}$$

since *CaS* and *CaT* are the only conductances that flux $[Ca^{2+}]$ into the cell. Here, *m* and *h* can be replaced by their steady state values $m_{\infty }$ and $h_{\infty }$ that are simply functions of *V*, since we assume that ${\dot{V}}=0$. Here $V_{Ca}$ is the value of the membrane potential where the calcium ODE has a zero.

Similarly, since we know ${\dot{V}}(V)$ (using Eq. (2)), we can solve for $V_V$, the value of the membrane potential where the voltage ODE has a zero. In addition, we can compute the marginal stability of this fixed point

9 $$\begin{aligned} \frac{\partial {\dot{V}}}{\partial V}=\frac{\partial }{\partial V}\sum _{i}{\bar{g}}_{i}m_{i}^{p}h_{i}^{q}(V-E_{i}) \end{aligned}$$

Now we impose the condition $V_V=V_{Ca}$ at every point in the space of conductance densities and find points where this equation is satisfied.

### Computing calcium level sets and basins of attraction

We defined a ‘calcium level set’ as the set of all points along the plane where the time-averaged intracellular calcium concentration was equal to that of the original model. This level set is conceptually significant since it is independent of the kinetic parameters of the homeostatic regulation mechanism; yet is the set of points along which the dynamics of the homeostatic mechanism is quenched (since the average intracellular calcium concentration is equal to the target).

In principle, this level set can be found using brute-force methods where a model is simulated at every point along the plane. A more efficient way to find this level set is to adaptively sample the plane, increasing the density of sample points at regions close to the level set. In practice, we did this by initially randomly sampling a few points in the plane, and building a Delaunay triangulation of sampled points. A new point was chosen for sampling based on the largest triangle that contained at least one node above the target calcium level and at least one node below. This process was iterated till an acceptable mesh size was achieved. A similar sampling algorithm was used to find the basins of attraction of the four categories of voltage dynamics when homeostasis was allowed to change the conductance densities of the neuron after perturbation.

### Parameterising generic high-dimensional perturbations

To characterise generic perturbations in conductance density that are not constrained to a plane (as in Fig. 5), we measured the mean and standard deviation of all conductance densities as follows:

10 $$\begin{aligned} \mu _{perturbation}= & {} \left\langle \frac{g-g_{0}}{g_{0}}\right\rangle \end{aligned}$$

11 $$\begin{aligned} \sigma _{perturbation}= & {} \sqrt{\frac{1}{N-1}\sum _{i=1}\left( \frac{g-g_{0}}{g_{0}} - \mu _{perturbation} \right) ^{2}} \end{aligned}$$

### Building a database of neuron models with similar dynamics

Following earlier work that generated databases of neuron models^35^, we constructed a set of neuron models with similar intrinsic dynamics by randomly sampling points from a hypercube in the 8-dimensional space of conductance densities. For each point, a model was initialised using these maximal conductances and its intrinsic voltage dynamics checked to see if it fell within acceptable tolerances of a pre-defined target activity. We have made a toolbox to efficiently construct these databases in parallel publicly available at https://github.com/sg-s/neuron-db.

### Model implementation and code availability

All models were implemented in xolotl, a freely available neuron and network simulator^104^. Voltage and calcium equations were numerically integrated using the exponential Euler method^105^ using a time step of 0.05 ms.

Code to reproduce all figures in this paper is available at https://github.com/marderlab/size-compensation.
